# Supplementary figures and images for: Evaluation of Different Types of Stimuli in an Event-Related Potential-Based Brain–Computer Interface Speller under Rapid Serial Visual Presentation
Source: Sensors (Basel). 2024 May 22;24(11):3315. doi: 10.3390/s24113315 (PMC11174573; doi:10.3390/s24113315)

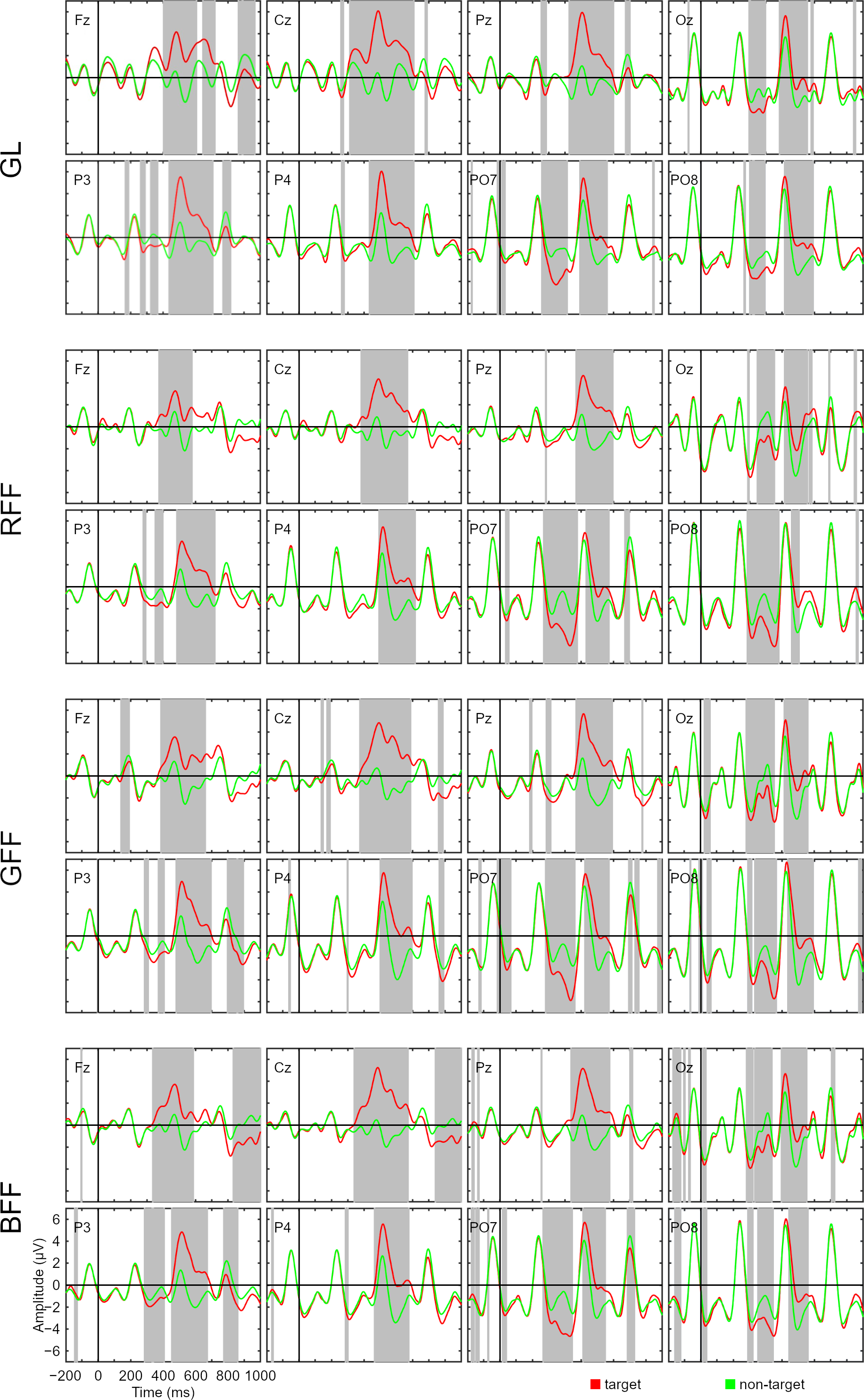

Supplement: Supplementary file 1 [file sensors-24-03315-s001.zip › figureS1.png]

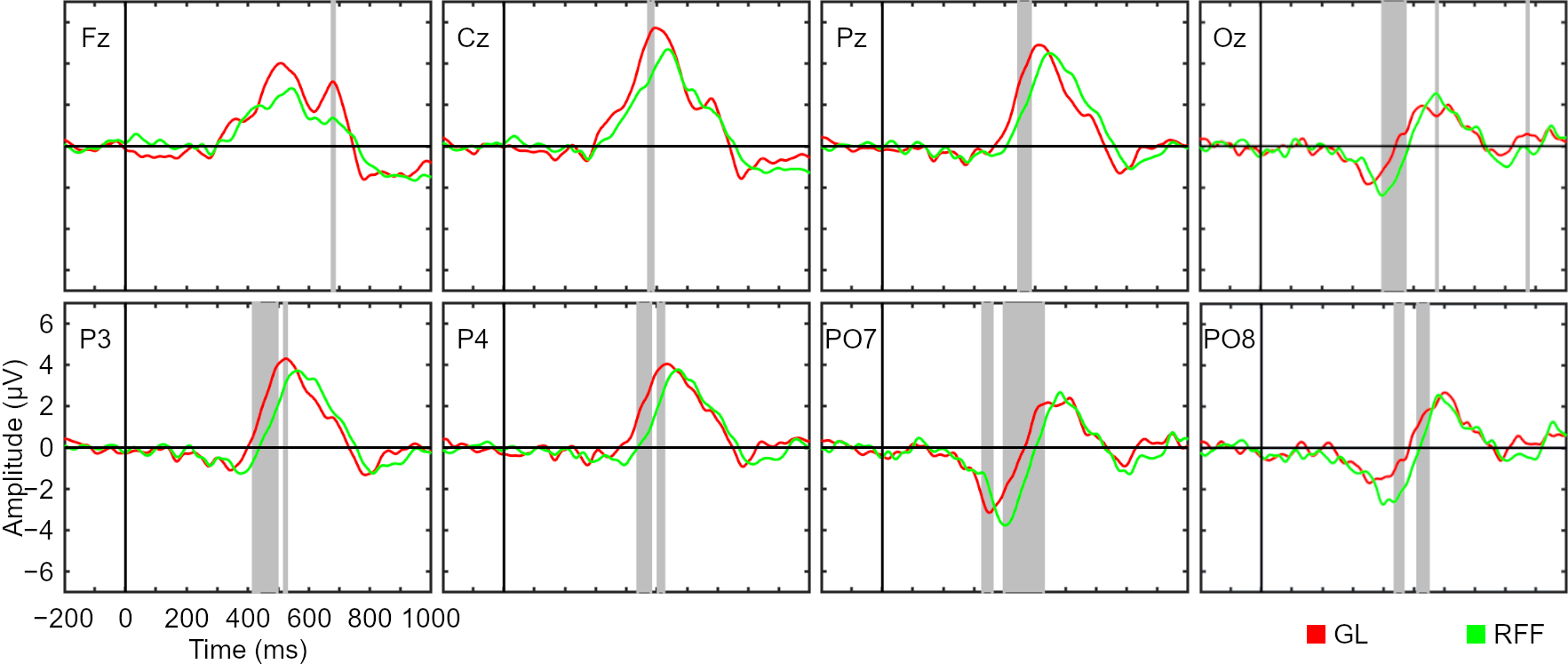

Supplement: Supplementary file 1 [file sensors-24-03315-s001.zip › figureS2.png]

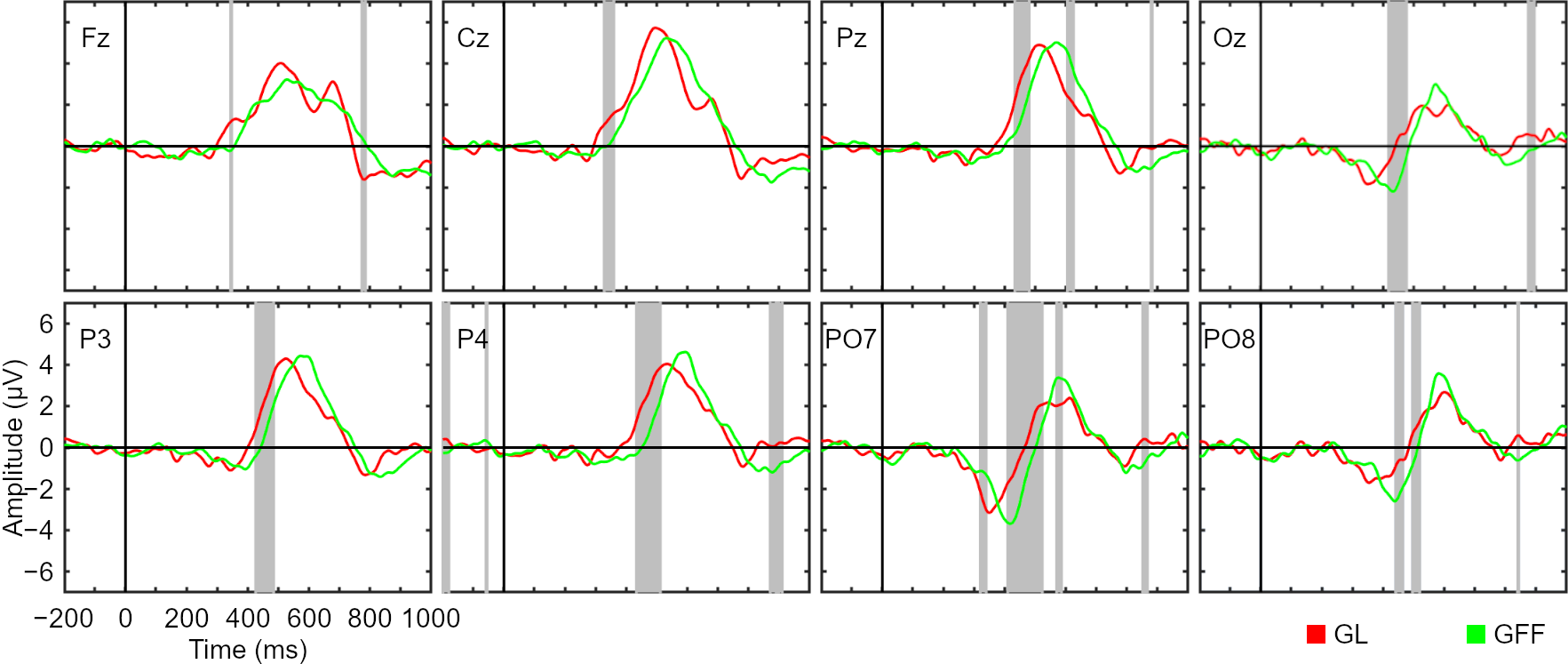

Supplement: Supplementary file 1 [file sensors-24-03315-s001.zip › figureS3.png]

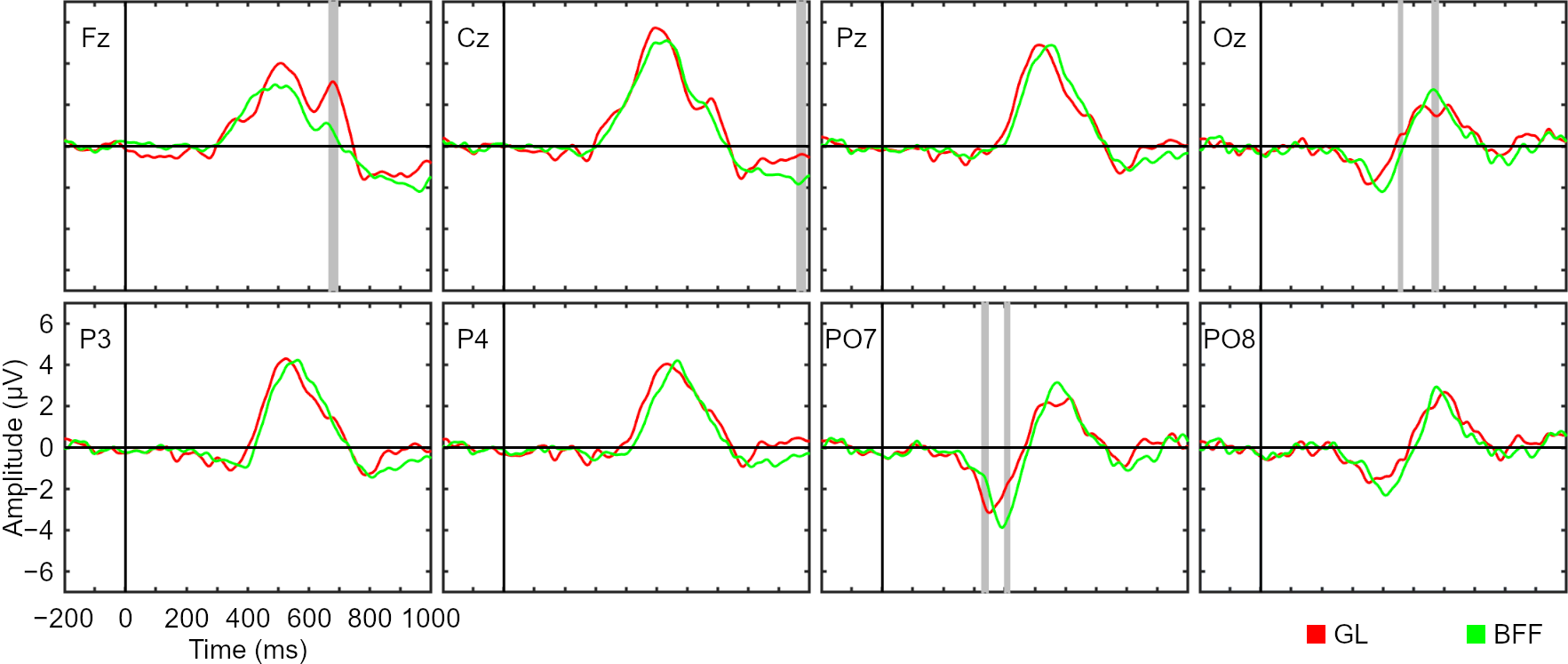

Supplement: Supplementary file 1 [file sensors-24-03315-s001.zip › figureS4.png]

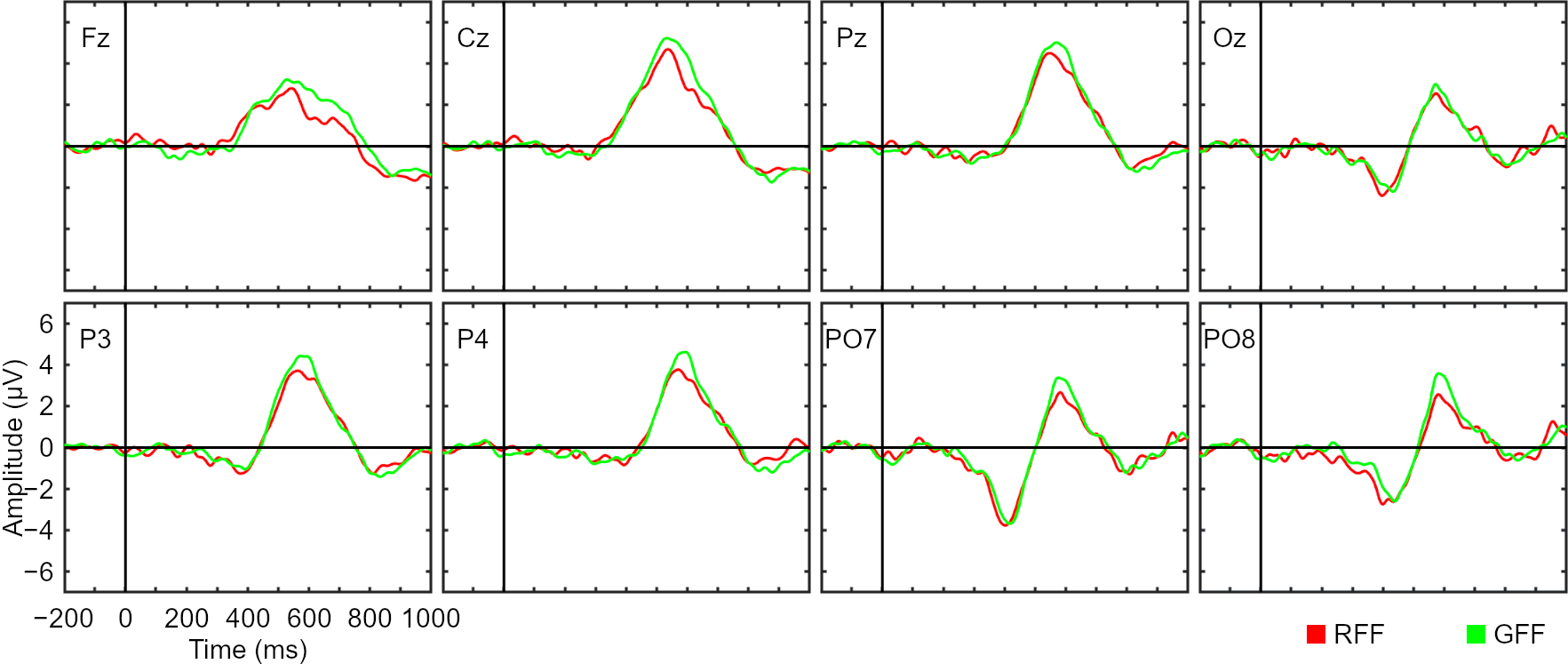

Supplement: Supplementary file 1 [file sensors-24-03315-s001.zip › figureS5.png]

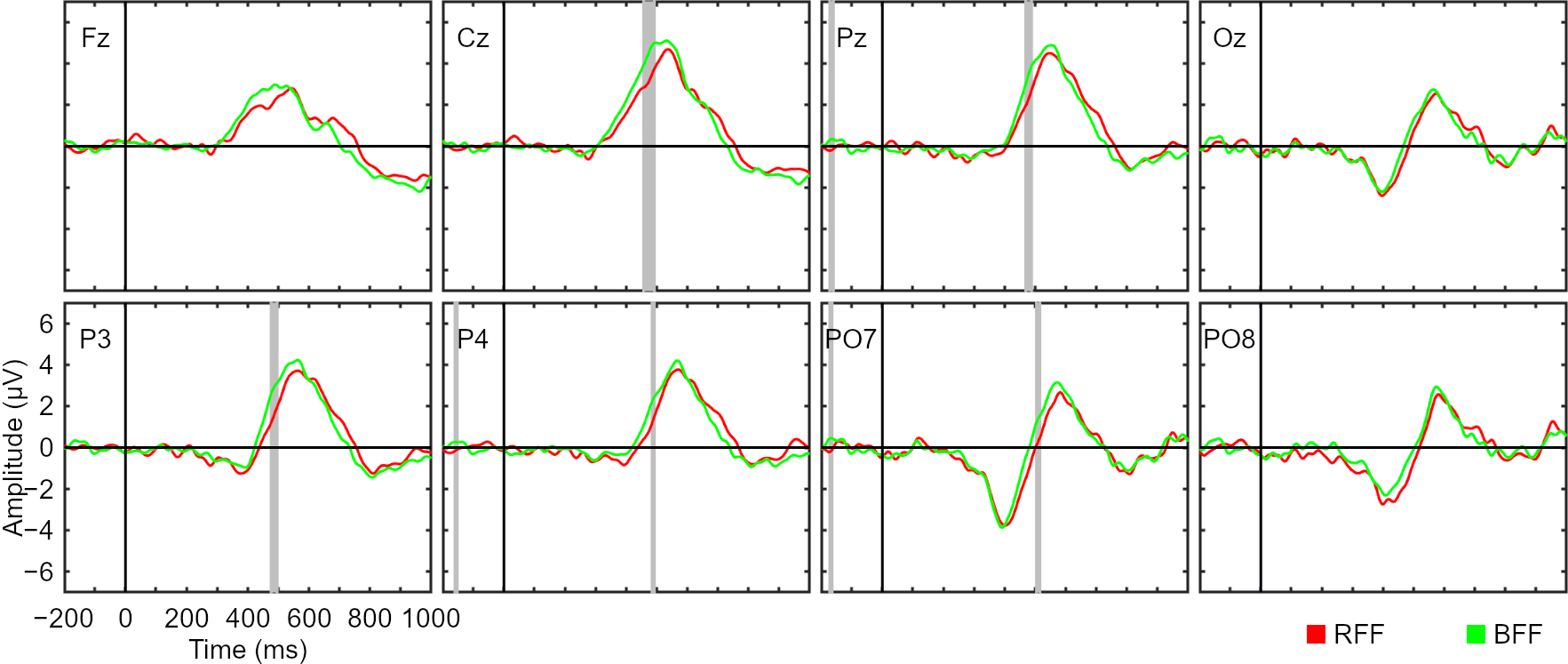

Supplement: Supplementary file 1 [file sensors-24-03315-s001.zip › figureS6.png]

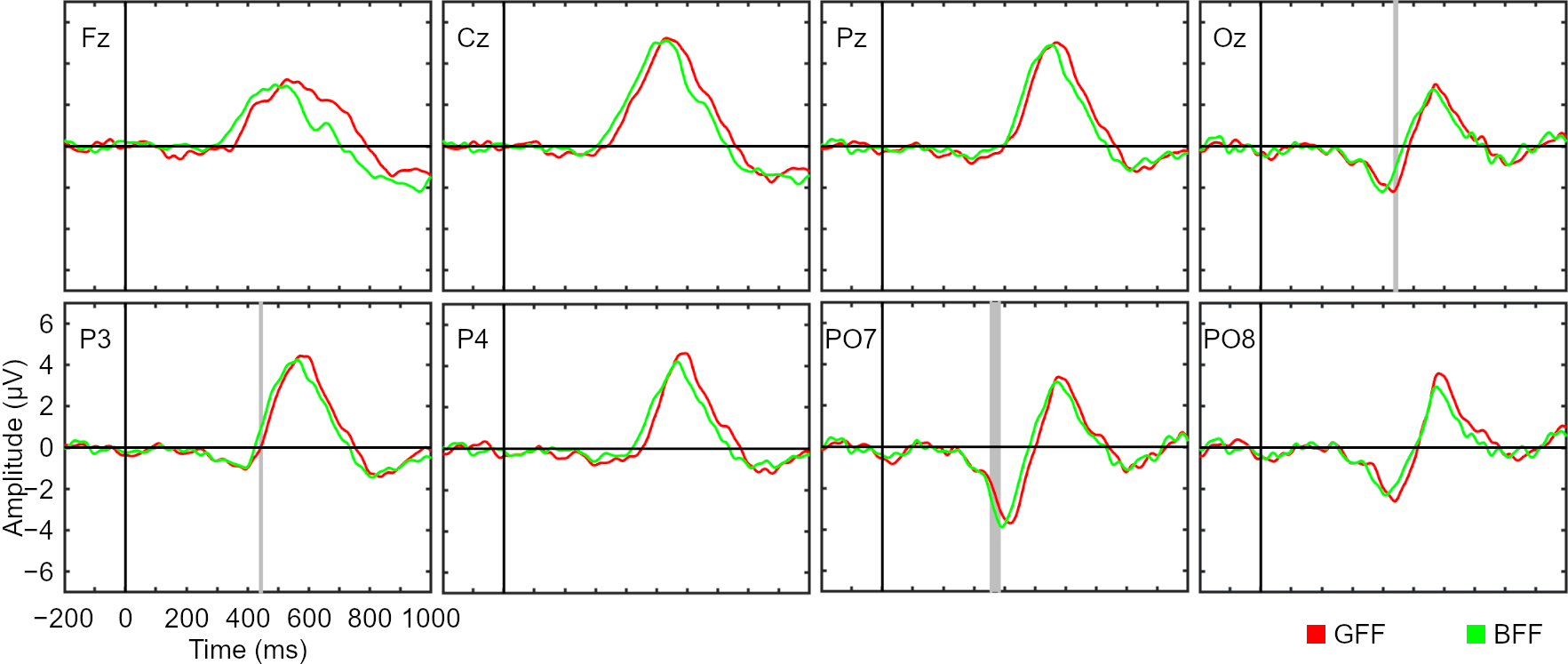

Supplement: Supplementary file 1 [file sensors-24-03315-s001.zip › figureS7.png]
